# Facts for the People

**Published:** 1868-07

**Authors:** E. Collins


### FACTS FOR THE PEOPLE.

BY E. COLLINS, D. D. S.

Messrs. Editors: Permit me to present to the readers of your journal a few briefly stated facts, which I trust will prove sufficiently interesting to be lastingly retained in the memory of every one, for it is by educating the public mind in matters pertaining to the curative professions that the practice of said professions are rendered not only the more pleasant and profitable to the practitioner in a pecuniary point of view, but profitable to the afflicted public in a manner that far transcends all pecuniary consideration.

In presenting the proposed facts, I shall do so by answering questions such as the following:

Can a tooth be saved after the nerve or pulp has become exposed?

If the nerve or pulp be deprived of its vitality with the appropriate agents and promptly removed from the tooth, and the root or roots be compactly filled with the proper material from point to margin of crown cavity, then I answer that about eighty per cent. of such teeth can be permanently saved. Can a tooth be saved after the parts investing the root or roots become inflamed, or after the inflammation has extended to one of its terminations, namely, suppuration?

If the Dentist will bear in mind that the tissues embracing the roots of the teeth are anatomically and physiologically identical with tissues in other parts, sustaining the same vital relations, and are subject to the same influences, both constitutional and local, that provoke and give character to inflammation in other organs or parts of the body, if the dentist would, with the above facts before him, be mindful to employ the same antiphlogistic remedies, general—where the symptoms indicate it—as well as local, as are employed in the treatment of inflammations elsewhere, then I answer that seventy-five per cent. of such teeth can be saved.

Can a tooth be saved after the crown has been one-half or two-thirds destroyed by decay?

If the remaining portion of the crown is dense and firm and freed from decay, and the root or roots are also cleansed out and medicated, a filling of the proper material can be anchored in the root or roots and in the remaining part of the crown, which latter can be restored to its original size, form, and usefulness.

The remaining per cent. of teeth that defies, in many cases, the skill of the Dentist, are almost exclusively found in the mouths of persons whose general recuperative energies have been, in a great measure, reduced by scrofulous, syphilitic, and mercurial diseases, and habitual intemperance in the use of alcoholic drinks, and the neglect, on the part of the patient, to duly respect the advice of him whom he has employed to treat his disease.

The above are obstacles not less frequently encountered by the dentist in the treatment of inflammations that come within the range of his specialty, than are encountered by other specialists, in the treatment of the same disease. Many an eminent Oculist, Aurist, general Surgeon, and Physician, have failed in many cases because of the above evils, and ever will; but because they fail they are not censurable, nor is it just to deny them credit and competent remuneration for their successes, which are far in the majority.

